# Do clinical data and human papilloma virus genotype influence spontaneous regression in grade I cervical intraepithelial neoplasia?

**DOI:** 10.4274/jtgga.2016.0138

**Published:** 2017-03-01

**Authors:** Caterina Cortés-Alaguero, Esteban González-Mirasol, José Morales-Roselló, Enrique Poblet-Martinez

**Affiliations:** 1 Department of Obstetrics and Gynecology, Complex Hospital of Albacete University, Albacete, Spain; 2 Department of Obstetrics and Gynecology, La Fe University and Polytechnic Hospital, Valencia, Spain; 3 Department of Pathological Anatomy, Reina Sofia General University Hospital, Murcia, Spain

**Keywords:** Human papilloma virus, cervical intraepithelial neoplasia grade I, human papilloma virus genotyping, regression, low-grade squamous intraepithelial lesion

## Abstract

**Objective:**

To determine whether medical history, clinical examination and human papilloma virus (HPV) genotype influence spontaneous regression in cervical intraepithelial neoplasia grade I (CIN-I).

**Material and Methods:**

We retrospectively evaluated 232 women who were histologically diagnosed as have CIN-I by means of Kaplan-Meier curves, the pattern of spontaneous regression according to the medical history, clinical examination, and HPV genotype.

**Results:**

Spontaneous regression occurred in most patients and was influenced by the presence of multiple HPV genotypes but not by the HPV genotype itself. In addition, regression frequency was diminished when more than 50% of the cervix surface was affected or when an abnormal cytology was present at the beginning of follow-up.

**Conclusion:**

The frequency of regression in CIN-I is high, making long-term follow-up and conservative management advisable. Data from clinical examination and HPV genotyping might help to anticipate which lesions will regress.

## INTRODUCTION

Cervical cancer and its precancerous lesions represent a major health issue, which needs early intervention in order to prevent high morbidity and mortality rates. Extensive research has revealed the existence of a close relationship between the onset of malignant lesions in the female tract and the presence of human papilloma virus (HPV) (1), especially regarding high oncogenic risk genotypes (2,3). The natural history of HPV infection indicates that following an initial HPV infection, a number of patients develop low-grade squamous intraepithelial lesions (LSIL), also known as cervical intraepithelial neoplasia grade I (CIN-I) (4), which occasionally progresses to high-grade squamous intraepithelial lesion (HSIL) (5,6) requiring exhaustive management and follow-up. However, the majority of CIN-I lesions regress without medical intervention, making treatment at this stage superfluous and cost-ineffective (7). Consequently, over-treatment at early stages should be avoided (8), especially in young women, and follow-up periods should be encouraged to make HPV clearance and histologic regression amenable (9).

The ability to identify patients whose lesions regress in advance would help to diminish costs, increase follow-up intervals, and decrease morbidity resulting from invasive diagnosis and unnecessary treatments (10,11,12,13). However, the means to anticipate this information are still unavailable. The aim of this study was to determine whether clinical information (medical history and examination) and HPV genotyping were useful to predict the frequency of regression and the need of treatment in CIN-I (14,15,16).

## MATERIALS AND METHODS

We retrospectively studied 232 women who were histologically diagnosed as having CIN-I at cervical pathology unit between 1995 and 2007. Most patients were referred for consultation because of an altered cytology. All women’s medical histories and clinical examinations including an initial cytology and colposcopy were evaluated. We took a biopsy in the first consultation to make the CIN-I diagnosis. The rest of the follow-ups were done using cytology. The patients were subsequently followed up every 6 months during the first two years, checking for the presence of progression, persistence or regression. When cytology results were normal, annual follow-ups were done for 5 additional years in some cases. CIN-I regression was defined as the disappearance of the lesion without treatment after two consecutive negative follow-ups, considering a negative follow up as both normal cytology and colposcopy, or in cases of altered colposcopy, negative follow up was considered as negative cytology and biopsy. If the cytology follow up resulted in HSIL, the biopsy was repeated. Progression was diagnosed if any change to HSIL was histologically detected. Finally, persistence was considered if no progression or regression was observed during the two-year follow-up. In case of persistence, appropriate treatment was applied.

Unfortunately, being a retrospective analysis, many exceptions applied. These circumstances determined the method of analysis, using Kaplan-Meier curves and excluding contingency tables to avoid comparison biases among the different clinical situations and HPV genotypes. A number of persistent lesions were followed up without treatment for more than 2 years and were seen to regress months afterwards. According to the Kaplan-Meier analysis, these cases were considered “still alive” at the end of the study and were consequently censored data (value=0). Some lesions were precipitately treated in the first or second year of follow-up and we therefore disregarded whether they regressed afterwards. According to the Kaplan-Meier analysis, these cases were considered as cases that “dropped off the study” and were consequently again treated as censored data (value=0). A third situation was when lesions progressed within the two-year follow-up interval and were treated. We considered them in the same group as those that dropped off the study, and were also classified as censored data (value=0). The time value in months assigned to these censored data was either the month of treatment or the limit of the study in case of follow-up for more than 2 years. Kaplan-Meier curves evaluated the pattern of regression using the log-rank (Mantel-Cox) test and also provided the median survival and hazard ratio with their 95% confidence intervals.

Regarding HPV, we evaluated the frequency of the different HPV genotypes (6, 11, 16, 18, 31, 33, 51, and 53) and also the different combinations of HPV genotypes (low- and high-risk HPV, single and multiple HPV). Regression was also evaluated in women with different clinical characteristics including age (<25, 25-34, 35-44, 45-54, and ≥55 years), menopause, age of first intercourse, parity, cigarette smoking, oral contraceptive use, condom use, of intrauterine device (IUD) use, results of the cytologic and colposcopic examination, and cervical extension of colposcopic findings. 

Exclusion criteria were: pregnancy, immunodeficiency, existence of concurrent vaginal lesions, follow-up less than two years, lack of data in the patient files, inability to obtain DNA, and treatment immediately after the diagnosis of CIN-I.

HPV genotype was obtained by means of polymerase chain reaction (PCR) according to earlier descriptions using a commercially available kit, SPF10 primers and line probe assay detection system (INNO-LiPA) HPV Genotyping Extra Amp. (Innogenetics, Ghent, Belgium). This system allowed the identification of 9 low-risk HPV genotypes (6, 11, 34, 40, 43, 44, 54, 70, 71), 16 high-risk HPV genotypes (16, 18, 31, 33, 35, 39, 45, 51, 52, 56, 58, 59, 68, 69, 73, 82) and 3 probable high-risk HPV genotypes (26, 53, 66). The kit also determined additional HPV genotypes (69/71/74) and unclassified HPV genotypes (types X).

The study was undertaken with a protocol authorized by the hospital. Statistics and graphs were constructed using Graph Pad Prism 5.0 (Graph Pad Software, La Jolla, CA, USA). Statistical significance was established at p<0.05.

## RESULTS

A description of the studied population at the onset of follow-up is shown in Table 1. A total of 232 women were included with a mean age of 34.6 years. Of them, 15 (6.5%) were menopausal, 55 (23.7%) had their first sexual intercourse prior to age 18 years, and 140 (60.3%) had delivered at least once. The most frequent family planning method was the condom, which was used by 88 women (37.9%), followed by hormonal contraception used by 47 women (20.2%), surgical methods 30 (12.9), and IUDs 19 (8.2). The majority of patients (n=218, 94%) were referred due to an abnormal cytology in the absence of any other symptoms, but only 162 (69.8%) presented with this finding at the onset of follow up. Other patients presented with vaginitis (4.3%), postmenopausal bleeding (0.4%) or intercourse bleeding (1.3%). Nearly all patients had abnormal colposcopy (94.4%), 68.1% of which was due to low risk changes. Finally, half of the patients (59.5%) smoked.

HPV infection was found in 224 patients (96.5%). Of these, 212 (91.4%) presented one or several high-risk HPV, 68 (29.3%) had one or several low-risk HPVs, and 4 (1.7%) additional or X HPV. Multiple HPV genotypes were found in 125 women (53.9%) with the following distribution: two different HPV genotypes in 81 women (34.9%), three in 25 (10.8%), four in 11 (4.7%), five in 6 (2.6%), seven in 1 (0.4%), and eight in 1 (0.4%). Table 2 shows the percentage of infection caused by each virus, isolated or combined with other HPV genotypes. The three most frequent high-risk HPV genotypes were HPV 16, 51, and 53. In 99 (42.7%) women, the etiology of infection was HPV 16. Of those, 38 (16.4% of the total number) presented with HPV16 as the only viral genotype. For HPV 51, this occurred in 46 (19.8%) and 18 (7.7%) cases, and for HPV 53 in 44 (19%), and 8 (3.4%) cases. In 12 women (5.2%), both HPV 16 and 18 were found. HPV 45 was found in only 2 (0.9%) women and always in association with other viral genotypes. In 99 (42.6%) women, HPV infection was caused by other high-risk viruses different to HPV 16 and 18. Finally, the most frequent low-risk virus was HPV 11 and less frequently, HPV 6.

Of the 232 CIN-I lesions, 116 regressed in the 2-year follow-up and 9 reached the end of the interval without regression. In addition, 93 were precipitately treated within the two years of follow-up and 14 progressed to HSIL and were treated during the follow-up interval. These last three groups were considered as censored data in Kaplan-Meier analyses.

**Figure 1:**
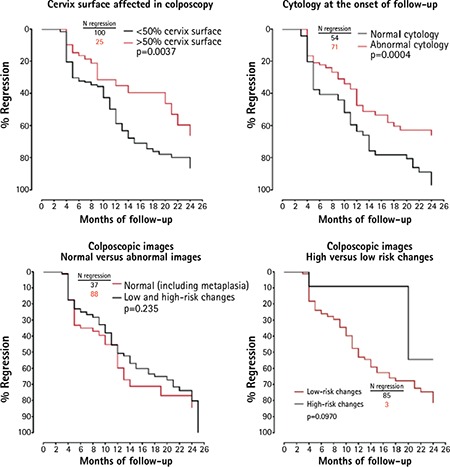
Kaplan-Meier curves evaluating the regression frequency of the cervical intraepithelial neoplasia grade I infections according to the percentage of cervical surface affected, the existence of an abnormal cytology at the onset of follow up, and the presence of different images (normal versus abnormal and high- versus low-risk changes) in the colposcopic examination

The analysis of the clinical parameters (Figure 1) showed that the regression frequency was significantly lower only in women who had an abnormal cytology at the onset of follow-up or who showed colposcopical abnormalities on more than 50% of the cervical surface. However, there was large influence on regression of characteristics related with sexuality such as condom use and age at first sexual intercourse, but it did not reach significance (p=0.0565 and p=0.0741). Influence of other characteristics such as age, colposcopical images (high- versus low-risk changes and normal versus abnormal findings), contraceptives use, IUD use, parity, menopause and smoking did not reach statistical significance. Comparison statistics are described in Table 3.

**Figure 2:**
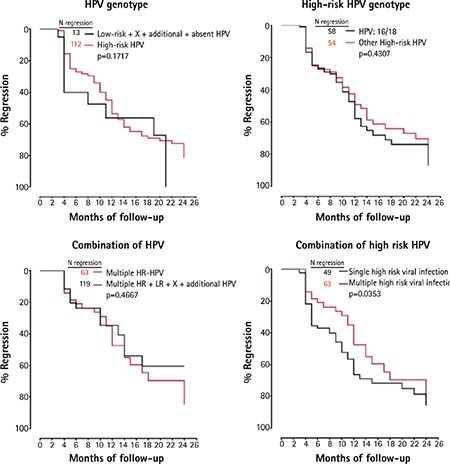
Kaplan-Meier curves evaluating the regression frequency of cervical intraepithelial neoplasia grade I infections according to the human papilloma virus genotype: high-risk versus low-risk, high-risk versus other high-risk, multiple high-risk versus any other multiple and finally single versus multiple high-risk human papilloma virus infection

With regards to the HPV genotypes, the analysis showed that regression frequency was significantly influenced only in the presence of multiple combinations of high-risk HPV (p=0.0353). Neither the presence of high-risk HPV versus low-risk HPV (p=0.1717) or the different combinations of high-risk HPV (p=0.4307) or the sum of any low-risk plus any high-risk HPV (p=0.4667) were seen to influence regression (Figure 2). Comparison statistics are also described in Table 3. The probabilities of CIN-I regression in relation to each HPV genotype, HPV16/18 or HPV 16/18/31/33/35/51/53/45/52/58 are shown in Table 4.

## DISCUSSION

Our results show that most of the CIN-I lesions disappeared in the two-year follow-up period, which is in line with earlier works quoting similar percentages (17, 18, 19, 20). Although HPV is the etiology of cervical cancer and intraepithelial neoplasia, the importance of the different viral genotypes is unclear (21). According to our data, 96.6% of CIN-I had HPV infection, HPV 16 being the most frequent genotype. This agreed with earlier investigations although with slightly lower percentages (22, 23). Also, although HPV 18 was the world’s second most frequent HPV genotype (24), it represented only 11.2% of our cases, a lower frequency than that of HPV 51 (19.8%), HPV 53 (19%), and HPV 31 (13.8%), in line with other works (25, 26).

Our results showed that HPV genotype (including HPV 16 and 18) was not a determinant of regression frequency as the survival curves did not show significant differences either in the pattern of regression between HPV 16/18 and the remaining high risk HPV or in the pattern of regression between high and low risk HPV. Concerning earlier references, very few studies examined the influence of the viral type in relation with regression. In addition, previous results were unclear. Some works showed a higher progression to CIN-III for HPV 16 (27), a higher progression to CIN-III and cervical cancer for HPV 16 (28) or a higher progression to CIN-III for HPV 16, 18, 31, 33, 35, 45, 52, and 58 (29). Conversely, others showed no increase to CIN-III for HPV 16/18 when compared with other high-risk HPV (30).

Regarding the combination of different HPV, our analysis showed that the number of high-risk viruses present in the epithelium (single or multiple high-risk HPV infections) influenced the frequency of regression. This was not in line with some previous works (31), but agreed with others that found differences, especially when one of the viruses was HPV 16 (32, 33, 34).

The method by which the sample was obtained (cytology or biopsy) and the PCR method applied are also important issues, because they could influence the number of HPV genotypes detected (35, 36). We applied SPF10 primers, genotyping with PCR-SPF10/LiPA. This was an excellent method to detect single and multiple HPV infection in paraffin fixed tissues (37, 38, 39), thanks to which we were able to identify HPV in a very high percentage of cases.

In consideration to clinical parameters, it has been suggested that smoking, use of oral contraceptives, sexual behavior, parity, and age at first sexual intercourse had an influence on the natural history of HPV intraepithelial lesions. However, most studies dealt with the influence on progression and very few on the influence on regression. Our data, in line with earlier works (27), showed a high influence of the onset of sexual intercourse or the use condom during sexual relationships, which may have yielded significant results with a slightly higher number of patients.

Although colposcopy is an excellent method to investigate abnormal cytologies (40), it cannot substitute histologic evaluation. Our data did not support an influence of colposcopy findings (suggesting lower or higher grade lesions) on the frequency of regression. However, similar to earlier works (41), the presence of a more extended lesion (>50% of the cervix surface) indicated that this was less likely to regress. Finally, we showed that regression was less frequent when anomalies were present in the initial cytology. Similar results were obtained in earlier studies, which showed a higher progression to CIN-III in these cases (42).

Finally, several strengths and limitations in the study should be underlined. Concerning the strengths: 1- We used only histologic diagnosis (a biopsy indicating CIN-I) avoiding the high false positive rate of cytology. 2- Our progression reference was CIN-III, avoiding the low correlation among pathologists in CIN-II lesions (43, 44). 3- The PCR method applied allowed a wide identification of HPV genotypes and the detection of multiple HPV infections with high sensibility, specificity, and celerity. The number of cases was also low. In addition, many lesions were precipitately treated prior to end of follow up, and therefore, the information of what would have happened if the follow-up had continued was unavailable.

In summary, most CIN-I lesions tend to a spontaneously resolve in the first two years of follow-up. However, the existence of abnormal cytology, an infection in more than 50% of the cervical surface or the presence of multiple viral high-risk HPV genotypes might influence the frequency of regression making this less likely.

## Figures and Tables

**Table 1:**
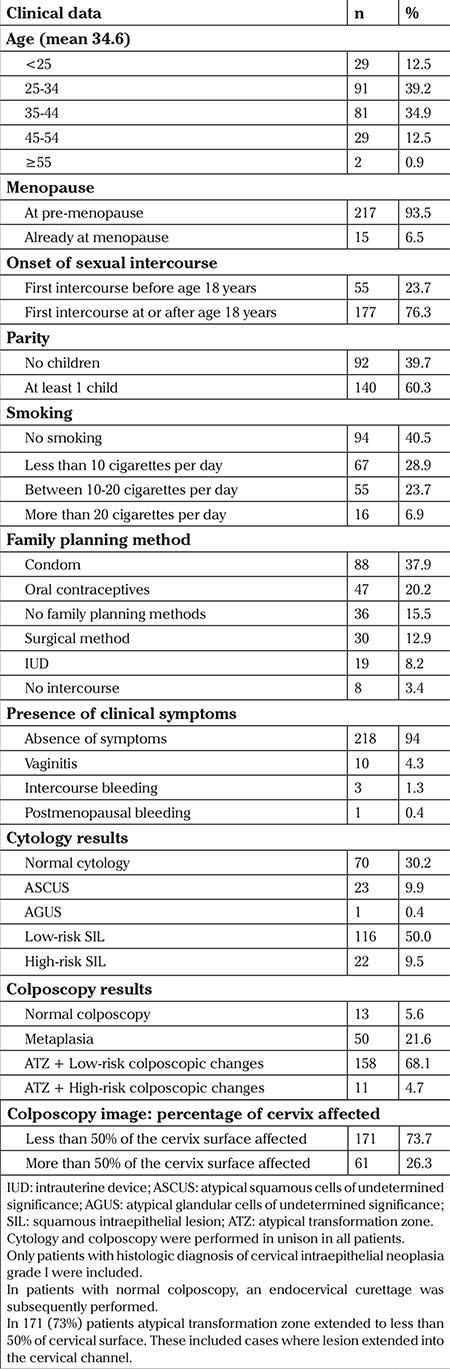
Clinical description of the population with histologic diagnosis of cervical intraepithelial neoplasia grade I

**Table 2:**
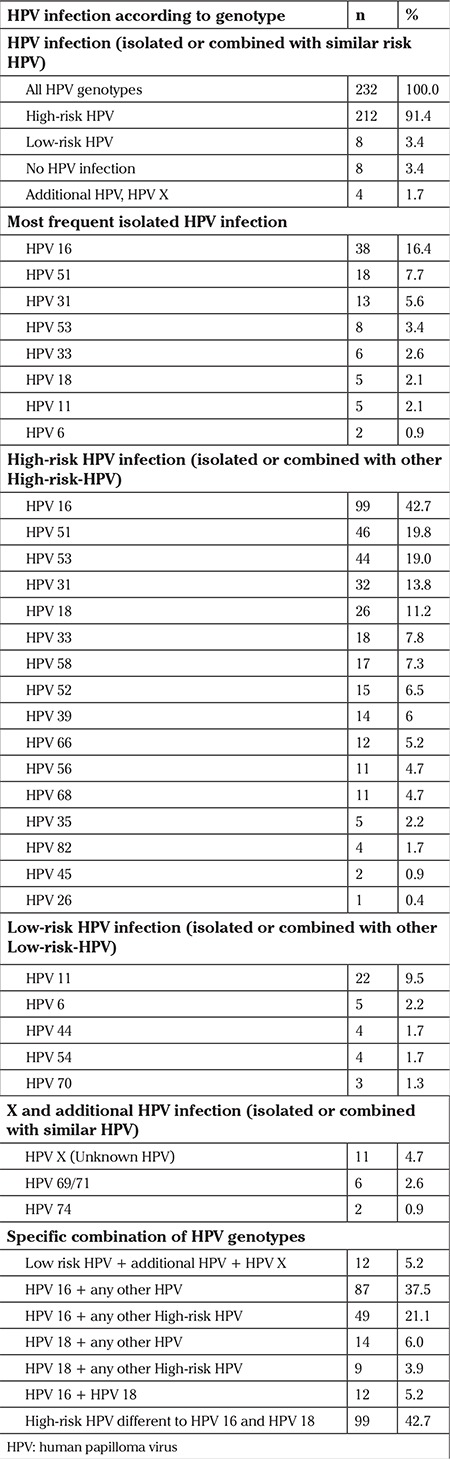
Distribution of human papilloma virus infection. n may reflect, isolated or combined human papilloma virus genotypes

**Table 3:**
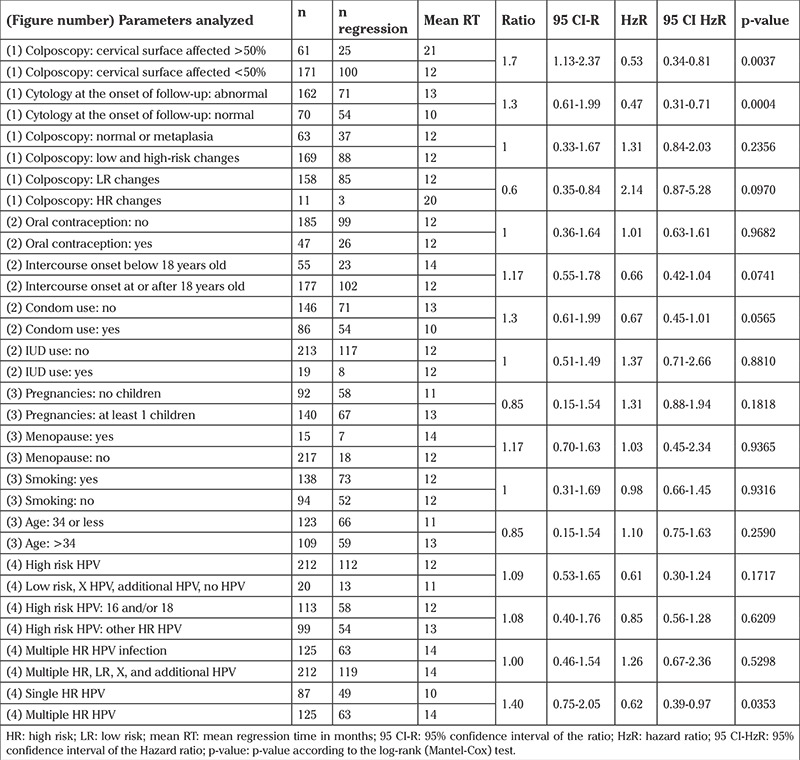
Kaplan-Meier statistics for the different parameters analyzed in Figures 1, 2

**Table 4:**
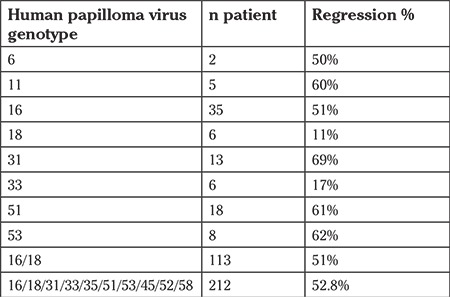
Probabilities of cervical intraepithelial neoplasia grade I regression in relation to human papilloma virus genotypes
